# Continuous Glucose Monitoring Metrics in High-Risk Pregnant Women with Type 2 Diabetes

**DOI:** 10.1089/dia.2023.0300

**Published:** 2023-11-23

**Authors:** Anna McLean, Elizabeth Barr, Georgina Tabuai, Helen R. Murphy, Louise Maple-Brown

**Affiliations:** ^1^ Wellbeing and Preventable Chronic Disease Division, Menzies School of Health Research, Charles Darwin University, Darwin, Australia.; ^2^ Endocrinology Department, Cairns Hospital, Cairns, Australia.; ^3^ Norwich Medical School, University of East Anglia, Norwich, United Kingdom.; ^4^ Endocrinology Department, Royal Darwin Hospital, Darwin, Australia.

**Keywords:** Glucose metrics, Flash glucose monitoring, Continuous glucose monitoring, Type 2 diabetes, Pregnancy, Indigenous, Aboriginal and Torres Strait Islander

## Abstract

**Objective:**

To describe glucose metrics in a high-risk population of women with type 2 diabetes (T2DM) in pregnancy and to explore the associations with neonatal outcomes.

**Research Design and Methods:**

Prospective observational study of 57 women. Continuous glucose monitoring (CGM) trajectories were determined from metrics collected in early and late gestation using the first and last two (mean 16 and 35) weeks of Freestyle Libre data. Logistic regression was used to examine associations of CGM metrics with neonatal hypoglycemia (glucose <2.6 mmol/L requiring intravenous dextrose) and large for gestational age (LGA) (>90th percentile for gestational age and sex). Pregnancy-specific target glucose range was 3.5–7.8 mmol/L (63–140 mg/dL).

**Results:**

Forty-one women used CGM for 15 weeks (mean age 33 years, 73% Aboriginal or Torres Strait Islander, 32% living remotely). There was limited change in average metrics from early to late pregnancy. For the subgroup with sensor use >50% (*n* = 29), mean time in range (TIR) increased by 9%, time above range reduced by 12%, average glucose reduced by 1 mmol/L, and time below range increased by 3%. Neonatal hypoglycemia was associated with most CGM metrics, HbA1c and CGM targets, particularly those from late pregnancy. LGA was associated with hyperglycemic metrics from early pregnancy. Each 1% increase TIR was associated with a 4%–5% reduction in risk of neonatal complications.

**Conclusion:**

In this high-risk group of women with T2DM, CGM metrics only improved during pregnancy in those with greater sensor use and were associated with LGA in early pregnancy and neonatal hypoglycemia throughout. Culturally appropriate health care strategies are critical for successful use of CGM technology.

## Background

Increasing prevalence of type 2 diabetes (T2DM) worldwide has led to an escalation in pregestational diabetes in pregnancy and concern regarding the persistently high rates of adverse pregnancy outcomes in this group.^1^ T2DM prevalence is particularly high among young Indigenous women globally,^2^ and those who experience socioeconomic disadvantage, social determinants of health, and other obstetric risks such as hypertension, smoking, and obesity.^1,3–5^ Maternal hyperglycemia is one of the key modifiable risk factors for pregnancy outcomes and continuous glucose monitoring (CGM) technology is a promising tool for optimizing diabetes management.

The addition of real-time CGM in pregnancy has been shown to reduce neonatal hypoglycemia, large-for-gestational-age (LGA) infants, and neonatal intensive care admissions for women with type 1 diabetes (T1DM).^6^ Intermittently scanned CGM (isCGM) has been shown to be acceptable^7^ and safe,^8,9^ with seemingly similar efficacy for glucose control during pregnancy compared with self-blood glucose monitoring,^9^ although randomized controlled trial data are lacking.^10,11^ Studies using isCGM in pregnancy have not yet demonstrated improvements in pregnancy outcomes.^9,12^ Despite the lack of data, the popularity of isCGM has grown^10^ and women with both T1DM and T2DM have been using this device as it is user friendly,^8^ preferable to multiple day and night finger sticks,^7^ and is becoming more affordable.^5^

In 2019, the *International Consensus on Time in Range* acknowledged that more data are required to demonstrate how CGM metrics relate to and predict clinical outcomes.^13^ Currently, there are guidelines for women with T1DM pregnancy: target HbA1c levels in the first trimester (<6.5%, <48 mmol/mol) and third trimester (<6.1%, <43 mmol/mol); CGM target glucose range 3.5–7.8 mmol/L (63–140 mg/dL) with time spent in range >70% (i.e., >16.8 h/day); time above range (TAR) <25% (<6 h/day); time below range (TBR) <4% (<1 h/day); and glycemic variability % coefficient of variation (CV) <36%.^13^ There is emerging evidence that CGM metrics are associated with neonatal outcomes in T1DM pregnancy. As little as 5%–7% improvement in time in range (TIR) (1.2–1.6 h/day) is associated with reduced risk of LGA^6,10,14^ and neonatal hypoglycemia.^5^

Studies have reported trimester-specific associations for CGM metrics with outcomes,^15^ some showing associations in each trimester,^16^ the second trimester,^12^ second and third trimesters,^5,6,10^ no associations,^17^ or limited additional value over HbA1c^18^ to predict neonatal complications.

There are no specific evidence-based TIR guidelines for women with T2DM and few women with T2DM in pregnancy have been included in CGM studies to date.^5,11,19^ There is a distinct lack of data from socioeconomically deprived populations experiencing a high burden of disease, for women who arguably have the greatest need. Data are urgently required to assess benefits of CGM in marginalized populations,^5^ to further assess associations of CGM metrics with neonatal outcomes and to define appropriate targets for women with T2DM.^7^

Far North Queensland is a large geographical area (270,000 km), with a high percentage of residents identifying as Aboriginal or Torres Strait Islander peoples and has among the highest prevalence of T2DM in youth reported worldwide.^4^ The Cairns Hospital provides a government-funded diabetes in pregnancy clinical service for all women in the region, encompassing in-person and telehealth appointments and outreach visits to remote communities. In this context, we conducted a pilot feasibility study using isCGM in addition to usual care for women with T2DM in pregnancy.^7^ The aim of this analysis was to describe maternal glucose metrics in early and late pregnancy and examine associations of metrics with neonatal hypoglycemia and LGA, for high-risk pregnant women with T2DM.

## Methods

### Study design and participants

This was a prospective, single-center, observational pilot study, including all women age ≥18 years referred to the diabetes service with preexisting T2DM in pregnancy before 30 weeks of gestation, from August 2019 to March 2021. Exclusion criteria were T1DM, gestational diabetes, or a concomitant medical condition that could prevent study completion. Fifty-seven women were given the FreeStyle^®^ Libre™ 1 Flash Glucose Monitor (reader and sensors) for the duration of pregnancy, in conjunction with usual care. There were 45 women who used the CGM device for >2 weeks. Four women were excluded, two who birthed at another institution for medical reasons (first trimester HbA1c 9.6%, 7.1%) and two stillbirths at 20 and 35 weeks of gestation (first trimester HbA1c 9.5%, 9.0%). Forty-one women had CGM and neonatal data available and were included in this analysis.

The schedule of usual care included monthly multidisciplinary reviews (endocrinologist, obstetrician, diabetes educator, and dietitian) in the first half of pregnancy, increasing to fortnightly from 28 weeks and weekly from 36 weeks of gestation. Blood glucose levels were reviewed by phone, telehealth, or e-mail 1–2 weekly throughout pregnancy. The local diabetes educator or midwife would upload readings to the “Libre View” website when sensors were replaced every 2 weeks. For accuracy evaluation, women were asked to do a paired finger-stick and scan glucose on the Freestyle Libre reader as often as possible.

### Glucose metrics

Data have been analyzed from two time points, “early” and “late” pregnancy, which equate to the first two weeks sensors were used (early second trimester, mean (standard deviation [SD]) 16 (7) weeks of gestation, range 6–28 weeks) and the last two weeks sensors were used (third trimester, mean (SD) 35 (2) weeks of gestation, range 28–38 weeks) for each individual participant. There were two women who used sensors for the first time at 28 weeks, and one woman who used sensors for the last time at 28 weeks. Glycemic data were downloaded from the Libre View website. Venous HbA1c was measured once per trimester in local laboratories. “Early” HbA1c refers to the first HbA1c measured in pregnancy up to 28 weeks of gestation.

CGM metrics included the following: TIR, defined as a percentage of all time with CGM glucose values within the pregnancy-specific target range of 3.5–7.8 mmol/L (63–140 mg/dL); TAR 7.8 mmol (>140 mg/dL); TBR 3.5 mmol/L (<63 mg/dL); mean glucose; glucose SD; interquartile range (IQR); % CV; and glucose management indicator (GMI). CGM targets were based on the consensus TIR targets proposed for T1DM pregnancy.^13^ The TIR target 3.5–7.8 mmol/L (63–140 mg/dL) was >70% (16 h 48 min), TAR target <25% (6 h), TBR target <4% (1 h), and CV target <36%. GMI targets were based on the HbA1c targets, <6.5% (<48 mmol/mol) in early and <6.1% (<43 mmol/mol) in late pregnancy. Treatment target glucose levels in addition to TIR were <5.1 mmol/L (<92 mg/dL) fasting, <7.8 mmol/L (<140 mg/dL) at 1 h, and <6.7 mmol/L (<121 mg/dL) at 2 h postprandially.

### Maternal characteristics and pregnancy outcomes

Maternal characteristics and pregnancy outcomes were measured prospectively and recorded in a Diabetes in Pregnancy Clinical Register.^20^ Maternal variables were age, measured first trimester body mass index (BMI), regional or remote (>100 km from Cairns) residence, self-identified ethnicity, parity, self-reported alcohol use and smoking in pregnancy, medical record of preexisting hypertension, use of metformin and/or insulin, and maximum recorded dose of insulin per day. Pregnancy outcomes were mode of delivery (cesarean section or vaginal birth), preeclampsia, prematurity (<37 weeks of gestation), respiratory distress (requiring oxygen supplementation), and neonatal length of stay in hospital. All babies born to women with T2DM are routinely admitted to the special care unit at our health facility, and so, this was not chosen as an outcome measure.

### Neonatal outcomes

Neonatal hypoglycemia was defined as blood glucose <2.6 mmol/L requiring intervention with intravenous dextrose.^21^ LGA was defined as >90th percentile for gestational age at birth and sex.^22^

### Statistical analyses

Maternal characteristics, pregnancy outcomes, CGM metrics, and HbA1c were described for the whole cohort and for infants with and without LGA and infants with and without neonatal hypoglycemia. Glucose metrics were compared from early to late pregnancy. Comparisons used a χ^2^ test or Fisher's exact test (categorical variables), independent or paired *t*-test, or Wilcoxon rank sum test if nonparametric (continuous variables). Continuous variables are given as mean (SD) if normally distributed or median (IQR). Unadjusted associations of CGM metrics and HbA1c measured in early and late pregnancy with the neonatal outcomes were assessed with logistic regression. Adjusted odds ratios and 95% confidence intervals were calculated using multivariable logistic regression adjusting separately for BMI, early HbA1c, and early TIR. Collinearity between the glucose metrics and these covariables was assessed using Pearson's *r* or Spearman's rho correlation coefficients.

Sensitivity analyses were performed to (1) describe glucose metrics and HbA1c measured in the first, second, and third trimesters and (2) describe glucose metrics among a subset of women who had sensor activity time >50% measured in the third trimester. Activity time of 50% was based on the distribution of the data, to ensure enough participants with “greater” sensor use for subgroup analysis. Statistical analyses were performed using Stata 15.1 (StataCorp, TX).

### Ethics

The study was approved by the Far North Queensland Human Research Ethics Committee with individual written patient informed consent.

## Results

### Maternal characteristics and pregnancy outcomes

The average age of women was 33 years, 73% of women identified as Aboriginal or Torres Strait Islander, 32% lived remotely, and median first trimester HbA1c was 7.8% (62 mmol/mol) (Table 1). On average, women used CGM for 15 weeks and scanned 4.4 times per day. Fifty-one percent of infants had neonatal hypoglycemia requiring intravenous dextrose and 56% were classified as having LGA. Neonatal hypoglycemia and LGA were characterized by less favorable maternal characteristics and pregnancy outcomes (Supplementary Table S1). Neonatal outcome groups were not significantly different in terms of mean gestation sensors were commenced or weeks of use, however, the time sensors were active in early pregnancy was less in those with LGA (*P* = 0.04) and neonatal hypoglycemia (*P* = 0.06) (Supplementary Table S1).

**Table 1.** Maternal Characteristics, Continuous Glucose Monitoring Use, and Pregnancy Outcomes

Maternal characteristics	Total* n* = 41
Age, years	33.2 (5.5)
First trimester body mass index, kg/m^2^	32.8 (5.9)
First trimester HbA1c, %	7.8 (6.6, 9.1)
Remote locality, *n* (%)	13 (32%)
Aboriginal or Torres Strait Islander ethnicity,^a^ *n* (%)	30 (73%)
Nulliparity, *n* (%)	6 (15%)
Preexisting hypertension, *n* (%)	8 (33%)
Smoking, *n* (%)	17 (43%)
Alcohol, *n* (%)	6 (16%)
Time since diagnosis <5 years, *n* (%)	15 (50%)
Insulin use, *n* (%)	37 (90%)
Insulin dose per day, units	89 (78)
Metformin use, *n* (%)	27 (66%)
CGM use	
Weeks of sensor wear	15.4 (7.8)
Sensor activity time in early pregnancy,^b^ %	63 (23)
Sensor activity time in late pregnancy,^b^ %	62 (20)
No. of scans per day in early pregnancy	4.3 (4.4)
No. of scans per day in late pregnancy	4.4 (3.1)
Gestation first used, weeks	16.0 (7.0)
Gestation last used, weeks	35.6 (2.2)
Pregnancy outcomes	
Prematurity, *n* (%)	14 (34%)
Gestational age at birth, weeks	37.4 (36, 38)
Birth weight, g	3497 (779)
Cesarean section, *n* (%)	27 (66%)
Preeclampsia, *n* (%)	8 (31%)
LGA >90th percentile, *n* (%)	23 (56%)
Neonatal hypoglycemia, *n* (%)	21 (51%)
Neonatal hospital stay, days	3 (2, 13)
Respiratory distress, *n* (%)	15 (37%)

Data are presented as mean (SD) or median (IQR). Total *n* is less for the following characteristics: first trimester HbA1c, *n* = 30; preexisting hypertension *n* = 24; alcohol use *n* = 37; time since type 2 diabetes diagnosis *n* = 30; early gestation sensor activity time and average scans per day in early pregnancy and early average scans per day, *n* = 38, preeclampsia *n* = 26.

^a^ Ethnicity is self-reported. Other ethnicities included Indian (*n* = 4), African (*n* = 2), Caucasian (*n* = 1), Filipino (*n* = 1), Pacific Islander (*n* = 1), not specified (*n* = 2).

^b^ Sensor activity time is the percent of time with CGM output over a 14-day period.

CGM, continuous glucose monitoring; IQR, interquartile range; LGA, large for gestational age; SD, standard deviation.

### CGM metrics: early versus late pregnancy

**FIG. 1.**
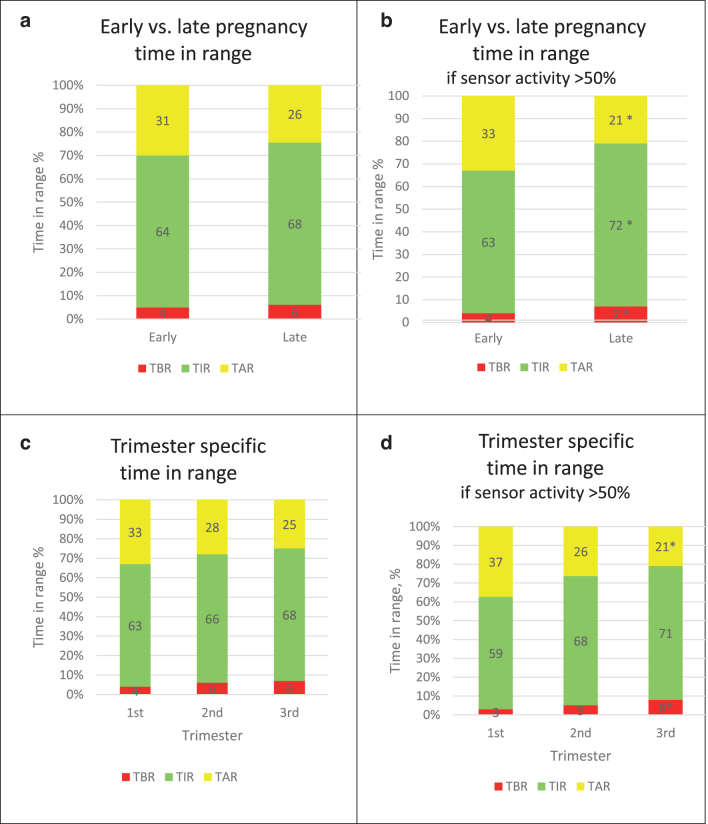
Mean percent TIR, TAR, and TBR in early versus late pregnancy and in each trimester. **(a)** Total cohort, *n* = 41, mean TIR% (SD), 64% (24) versus 68% (21), *P* = 0.41; TAR% (SD), 31% (26) versus 26% (22), *P* = 0.27; TBR% (SD), 4% (6) versus 6% (6), *P* = 0.25. **(b)** In those with sensor activity >50% in the third trimester, *n* = 29, mean TIR% (SD), 63% (25) versus 72% (16), *P* = 0.04; TAR% (SD), 33% (27) versus 21% (18), *P* = 0.01; TBR% (SD), 4% (4) versus 7% (7), *P* = 0.01. **(c)** First trimester *n* = 16, second trimester *n* = 32, third trimester *n* = 41. TIR% (SD) 63% (21), 66% (22), 68% (21), *P* = 0.9; TAR% (SD) 33% (24), 28% (23), 25% (22), *P* = 0.7 and TBR% (SD), 4% (3), 6% (8), 6% (7), *P* = 0.05 in each trimester, respectively. **(d)** First trimester *n* = 12, second trimester *n* = 23, third trimester *n* = 29. TIR% (SD) 59% (24), 68% (22), 71% (16), *P* = 0.08; TAR% (SD) 37% (25), 26% (23), 21% (17), *P* = 0.03; TBR% (SD) 3% (4), 5% (5), 8% (8), *P* = 0.04. Glucose target range defined as 3.5–7.8 mmol/L (63–140 mg/dL) and TIR, TAR, TBR expressed as a percent of all time with continuous glucose monitoring output over a 14-day period. Early pregnancy, first 2 weeks of sensor use, mean (range) gestation 16 (6–28) weeks. Late pregnancy, last 2 weeks of sensor use, mean (range) gestation 35 (28–38) weeks. SD, standard deviation; TAR, time above range; TBR, time below range; TIR, time in range.

There were no significant changes in mean TIR, TBR, and TAR from early to late pregnancy (Fig. 1a) or across each trimester (Fig. 1c) using pregnancy-specific target glucose levels 3.5–7.8 mmol/L (63–140 mg/dL). There were no significant changes in GMI, average glucose, or CV (Supplementary Fig. 1a). More favorable improvements in CGM metrics were observed for the subgroup of women with sensor activity >50% of the time (*n* = 29). This group showed a 9% increase in TIR, 12% reduction in TAR, 1 mmol/L reduction in average glucose, and a 3% increase in TBR and CV (Fig. 1b, d, and Supplementary Fig. S1b). Individual variability of TIR from early to late pregnancy is demonstrated in Supplementary Figure S2. There were no significant differences between early and late SD and IQR, or in the proportion of women achieving TIR targets (data not shown).

### CGM metrics and associations with neonatal hypoglycemia and LGA

Those with neonatal hypoglycemia had higher measures of early pregnancy hyperglycemia, including lower TIR, higher TAR, higher average glucose, and median GMI, compared with those without neonatal hypoglycemia. In late pregnancy, all metrics (except TBR) were significantly different between the groups, including hyperglycemia (lower TIR, higher TAR, average glucose, and GMI) and glucose variability metrics (higher SD, IQR, and CV). Lower proportions of women achieved the CGM targets in the group with neonatal hypoglycemia. This group also had higher median HbA1c and a lower proportion who achieved HbA1c targets in each trimester (Table 2 and Supplementary Table S4).

**Table 2.** Comparison of Continuous Glucose Monitoring Metrics and HbA1c Levels with Neonatal Hypoglycemia and Large for Gestational Age

CGM metrics and HbA1c	Total* n* = 41	Neonatal hypoglycemia* n* = 21 (51%)	No neonatal hypoglycemia* n* = 20 (49%)	*P*	LGA* n* = 23 (56%)	No LGA* n* = 18 (44%)	*P*
Early pregnancy metrics							
TIR, %	65 (24)	**58 (23)**	**73 (23)**	**0.05**	**57 (27)**	**75 (16)**	**0.02**
TAR, %	31 (26)	**39 (25)**	**21 (24)**	**0.03**	**40 (29)**	**19 (17)**	**0.01**
TBR, %	4 (6)	3 (4)	6 (9)	0.28	3 (3)	6 (9)	0.16
Average glucose, mmol/L	7.0 (1.9)	**7.6 (1.9)**	**6.3 (1.6)**	**0.03**	**7.6 (2.1)**	**6.2 (1.2)**	**0.01**
GMI, %	6.1 (5.7, 6.5)	**6.3 (5.9, 6.6)**	**5.8 (5.4, 6.3)**	**0.04**	6.3 (5.9, 6.6)	5.8 (5.5, 6.3)	0.09
SD, mmol/L	1.9 (0.6)	**2.1 (0.5)**	**1.6 (0.6)**	**0.01**	2.0 (0.4)	1.8 (0.7)	0.19
CV%	28 (6.9)	30 (7)	27 (7)	0.15	28 (6.4)	28 (7.8)	0.94
IQR	2.3 (0.6)	**2.8 (0.5)**	**1.9 (0.6)**	**0.001**	**2.7 (0.5)**	**2.0 (0.6)**	**0.006**
Early HbA1c, %	7.8 (6.5, 8.6)	**8.1 (7.6, 8.9)**	**6.4 (5.7, 6.9)**	**0.001**	7.7 (6.8, 8.8)	7.0 (5.5, 8.3)	0.17
Early glycemic targets							
TIR >70%, *n* (%)	19 (50%)	**6 (30%)**	**13 (72%)**	**0.009**	9 (43%)	10 (59%)	0.32
TAR <25% *n* (%)	21 (55%)	**7 (35%)**	**14 (78%)**	**0.008**	9 (43%)	12 (71%)	0.08
TBR <4%, *n* (%)	22 (59%)	12 (60%)	10 (59%)	0.94	13 (62%)	9 (56%)	0.72
CV <36%, *n* (%)	30 (81%)	16 (80%)	14 (82%)	0.85	18 (86%)	12 (75%)	0.41
GMI <6.5, *n* (%)	26 (79%)	14 (74%)	12 (86%)	0.40	14 (74%)	12 (86%)	0.40
Early HbA1c <6.5%, *n* (%)	10 (26%)	**0**	**10 (56%)**	**0.000**	4 (18%)	6 (38%)	0.18
Late pregnancy metrics							
TIR, %	68 (21)	**60 (19)**	**77 (19)**	**0.007**	65 (18)	72 (25)	0.32
TAR, %	26 (22)	**34 (20)**	**15 (20)**	**0.007**	30 (19)	20 (25)	0.17
TBR, %	6 (6)	6 (7)	7 (7)	0.68	5 (5)	8 (8)	0.20
Average glucose, mmol/L	6.5 (1.4)	**6.9 (1.3)**	**5.9 (1.3)**	**0.01**	6.8 (1.3)	6.0 (1.3)	0.09
GMI, %	6.1 (5.7, 6.3)	**6.2 (6.0, 6.4)**	**5.7 (5.5, 6.2)**	**0.02**	**6.2 (5.7, 6.4)**	**5.8 (5.5, 6.3)**	**0.05**
SD, mmol/L	1.9 (0.6)	**2.2 (0.5)**	**1.7 (0.6)**	**0.005**	**2.1 (0.5)**	**1.7 (0.6)**	**0.04**
CV%	30 (6.2)	**32 (6)**	**28 (6)**	**0.03**	32 (5.1)	28 (7.0)	0.22
IQR, mmol/L	2.7 (1.0)	**3.2 (0.9)**	**2.2 (0.7)**	**0.001**	**3.0 (0.9)**	**2.3 (0.9)**	**0.03**
Third trimester HbA1c, %	6.8 (6.1, 7.4)	**6.9 (6.3, 8.1)**	**6.3 (5.8, 7.1)**	**0.04**	**6.8 (6.5, 7.7)**	**6.1 (5.8, 7.1)**	**0.05**
Late glycemic targets							
TIR >70%, *n* (%)	24 (59%)	**7 (33%)**	**17 (85%)**	**0.001**	12 (52%)	12 (67%)	0.35
TAR <25%, *n* (%)	24 (60%)	**8 (38%)**	**16 (84%)**	**0.003**	11 (48%)	13(76%)	0.07
TBR <4%, *n* (%)	18 (44%)	11 (52%)	7 (35%)	0.26	11 (48%)	7 (38%)	0.56
CV <36%, *n* (%)	31 (76%)	**13 (62%)**	**18 (90%)**	**0.03**	17 (74%)	14 (78%)	0.77
GMI <6.1, *n* (%)	17 (50%)	**5 (28%)**	**12 (75%)**	**0.006**	**7 (35%)**	**10 (71%)**	**0.03**
GMI <6.5, *n* (%)	28 (85%)	15 (83%)	13 (87%)	0.79	16 (80%)	12 (90%)	0.33
Third trimester HbA1c <6.1%, *n* (%)	8 (23%)	**1 (6%)**	**7 (39%)**	**0.02**	**1 (6%)**	**7 (41%)**	**0.01**

Data are presented as mean (SD) or median (IQR) or *n* (%); total *n* is less for the following characteristics: Early HbA1c *n* = 38, third trimester HbA1c *n* = 35. Early metrics, first 2 weeks of sensor use, mean (range) gestation 16 (6–28) weeks. Late metrics, last 2 weeks of sensor use, mean (range) gestation 35 (28–38) weeks. Early HbA1c, first or second trimester HbA1c, mean (SD) gestation 9.6 (6) weeks. Range defined as glucose 3.5–7.8 mmol/L (63–140 mg/dL) and TIR/TAR/TBR expressed as a percentage of all time CGM is active over a 14-day period.

CV, coefficient of variation; GMI, glucose management indicator; TAR, time above range; TBR, time below range; TIR, time in range.

Those with LGA had lower TIR, higher TAR, and higher average glucose in early pregnancy compared with those without LGA. In late pregnancy, those with LGA had a lower proportion of women with HbA1c <6.1% (<43 mmol/mol) and GMI <6.1% (Table 2 and Supplementary Table S4).

Associations of HbA1c and CGM metrics with neonatal hypoglycemia and LGA are shown in Table 3. The metrics associated with both outcomes in early pregnancy were measurements of hyperglycemia (higher TAR, average glucose and IQR), and for both outcomes in late pregnancy were measurements of glucose variability (higher SD and IQR) and attainment of optimal glucose management targets (GMI <6.1% and third trimester HbA1c <6.1%, <43 mmol/mol). TBR and GMI <6.5% were not significantly associated with either outcome in early or late pregnancy. CGM metric associations were stronger in late pregnancy for neonatal hypoglycemia and in early pregnancy for LGA. Each 1% increase in TIR was associated with a 4%–5% reduction in the risk of LGA and neonatal hypoglycemia and a 1 mmol increase in average glucose nearly doubled the risk of LGA (early pregnancy) and neonatal hypoglycemia (late pregnancy).

**Table 3.** Unadjusted Associations for Continuous Glucose Monitoring Metrics and HbA1c with Neonatal Hypoglycemia and Large for Gestational Age

CGM metrics and HbA1c	Neonatal hypoglycemia		LGA	
	OR	95% CI	OR	95% CI
Early pregnancy metrics^a^				
TIR, %	0.96	0.93–1.00	**0.96**	**0.92–0.99**
TAR, %	1.04	0.99–1.06	**1.04**	**1.01–1.08**
TBR, %	0.93	0.83–1.05	0.91	0.80–1.04
Average glucose, mmol/L	**1.67**	**1.01–2.78**	**1.84**	**1.04–3.28**
GMI, %	3.07	0.92–10.2	2.58	0.83–7.91
SD, mmol/L	**4.91**	**1.26–19.0**	2.07	0.68–6.21
CV%	1.07	0.97–1.18	0.99	0.91–1.09
IQR, mmol/L	**26.7**	**2.41–293**	**6.48**	**1.36–30.7**
Early HbA1c, %	**1.61**	**1.01–2.54**	1.21	0.84–1.71
Early glycemic targets^b^				
TIR >70%	**0.16**	**0.04–0.67**	0.52	0.14–1.91
TAR <25%	**0.15**	**0.03–0.65**	0.31	0.08–1.21
TBR <4%	1.05	0.28–3.51	1.26	0.33–4.70
CV <36%	0.85	0.16–4.51	2.00	0.37–10.5
GMI <6.5	0.43	0.10–1.79	0.46	0.07–2.85
Early HbA1c <6.5% (<48 mmol/mol)	**—**	**—**	0.37	0.08–1.63
Late pregnancy metrics^a^				
TIR, %	**0.94**	**0.90–0.99**	0.98	0.95–1.01
TAR, %	**1.05**	**1.01–1.09**	1.02	0.98–1.05
TBR, %	0.98	0.90–1.07	0.94	0.85–1.03
Average glucose, mmol/L	**1.97**	**1.08–3.61**	1.57	0.91–2.69
GMI, %	3.90	0.85–17.7	3.35	0.73–15.3
SD, mmol/L	**5.64**	**1.47–21.5**	3.39	0.99–11.5
CV%	**1.13**	**1.01–1.26**	1.06	0.96–1.22
IQR, mmol/L	**5.25**	**1.71–16.1**	**2.29**	**1.02–5.10**
Third trimester HbA1c, %	2.01	0.91–4.65	1.59	0.83–3.05
Late glycemic targets^b^				
TIR >70%	**0.08**	**0.01–0.41**	0.54	0.15–1.92
TAR <25%	**0.11**	**0.02–0.52**	0.28	0.08–1.12
TBR <4%	2.04	0.58–7.17	1.44	0.41–5.03
CV <36%	**0.18**	**0.03–0.99**	0.80	0.18–3.44
GMI <6.1	**0.13**	**0.03–0.59**	**0.21**	**0.04–0.94**
GMI <6.5	0.78	0.11–5.33	0.33	0.03–3.37
Third trimester HbA1c <6.1% (<43 mmol/mol)	**0.09**	**0.01–0.91**	**0.08**	**0.01–0.78**

Bold denotes significance *p* < 0.05.

Data are OR and 95% CI. Early metrics, first 2 weeks of sensor use, mean (range) gestation 16 (6–28) weeks. Late metrics, last 2 weeks of sensor use, mean (range) gestation 35 (28–38) weeks. Early HbA1c, first or second trimester HbA1c, mean (SD) gestation 9.6 (6) weeks. Glucose target range defined as glucose 3.5–7.8 mmol/L (63–140 mg/dL) and TIR/TAR/TBR expressed as a percentage of all time CGM is active over a 14-day period.

^a^ Odds ratio per unit increase.

^b^ Odds ratio for meeting target.

CI, confidence intervals; OR, odds ratio.

Adjusting for BMI or early TIR did not attenuate the associations of CGM metrics with either neonatal outcome. When adjusted for early HbA1c, early and late targets TIR >70% and GMI <6.1% were still significantly associated with neonatal hypoglycemia but not with LGA (Supplementary Tables S2 and S3). Correlation coefficients between metrics and early HbA1c ranged from 0.2 to 0.7.

### CGM accuracy

The mean absolute relative difference (ARD) calculated from a total of 573 scanned versus capillary glucose measurements was 16.7% and the median ARD was 13.6% (Supplementary Fig. S3).

## Discussion

In this study involving high-risk women from regional and remote Australia, a key finding was the limited change in glucose metrics from early to late pregnancy. However, significant improvement in glucose trajectories was observed in the subgroup that had greater sensor activity time. Our study demonstrated alarming rates of neonatal complications, which were observed in the context of persistent maternal hyperglycemia in T2DM pregnancy. Neonatal hypoglycemia was associated with nearly all CGM metrics, HbA1c levels, and CGM target attainment in early and in late pregnancy. LGA was associated with maternal hyperglycemia in early pregnancy.

T2DM prevalence is particularly high among Indigenous populations globally,^2^ and is exacerbated in each generation by exposure to hyperglycemia in utero.^1,3,23^ In the third trimester, women in this study still spent over 5 h of the day above target (TAR 24%), with target glucose level 3.5–7.8 mmol/L (63–140 mg/dL). This is considerably higher than previously described in a U.K. study,^24^ where TAR reduced to 12% in the third trimester. The hyperglycemia metrics in our patient population were closer to those reported in T1DM pregnancies (third trimester TAR 27%,^6^ 34%^10^). This emphasizes the need for data from a variety of population groups to inform guideline recommendations.

Glucose levels in this cohort were similar to other studies of Aboriginal Australian women,^25^ but were higher compared with non-Indigenous cohorts with T2DM pregnancy, when comparing HbA1c^1,24^ and TIR.^24^ More consistent sensor use has been shown to improve glycemic levels^9^ and is supported by our findings that only the group with increased sensor activity time had improved glycemia throughout pregnancy.

Unlike in other studies,^6,24^ TBR increased by 3% from early to late pregnancy in our cohort, which may have been related to intensification of treatment over time. Other possible contributors include a component of increased insulin sensitivity and/or placental insufficiency in late pregnancy contributing to hypoglycemia in some women,^26^ or increased inaccuracy of the Libre 1 system throughout pregnancy. For accuracy evaluation, women were asked to do a paired finger-stick and scan glucose on the Freestyle Libre reader as often as possible. The mean ARD was higher (16.7%) in our study than others have reported.^8,27^ The CGM measurements had a slight negative bias, tending to read lower than the finger-stick measurements (Supplementary Fig. S3).

An increase in TBR may be a positive marker of improved glycemia as there are some data suggesting that TBR is inversely related to LGA^28^ and neonatal hypoglycemia risk.^29^ Whether to use a target of TBR <4% requires more research with newer generation sensors that have a higher accuracy in the low-glucose range.

Neonatal hypoglycemia was associated with all the glucose metrics except TBR and CV in early pregnancy and TBR in late pregnancy. In T1DM pregnancy, a modest increase of 5%–7% increase TIR has been associated with reduced risk of neonatal hypoglycemia.^21^ Our group with neonatal hypoglycemia had 15%–17% less TIR and 1–1.3 mmol/L (18–24 mg/dL) higher average glucose than those without neonatal hypoglycemia. Markers of glucose variability appeared to be associated with neonatal hypoglycemia in late pregnancy, consistent with findings from some,^30^ but not all, previous studies in T1DM.^18^ Proposed CGM targets^13^ TIR >70% and TAR <25% were significantly associated with a lower risk of neonatal hypoglycemia in both early and late pregnancy.

HbA1c is known to be predictive of neonatal hypoglycemia risk^21,31,32^ particularly in the second and third trimester. In our cohort, a higher HbA1c in each trimester was associated with greater risk of neonatal hypoglycemia. Achieving a GMI of <6.1% also showed an association with reduced neonatal hypoglycemia risk. It is unclear whether GMI could be used as a clinical treatment target, given the known discordance between GMI and HbA1c.^33^

LGA was associated with elevated early pregnancy average glucose and TAR, similar to others^10,12,28^ in T1DM pregnancy. A 1% higher TIR in early pregnancy was associated with 4% lower risk of LGA, similar in magnitude to others,^10,12^ supporting the need for optimizing early pregnancy TIR to reduce the risk of LGA, ideally before the end of the first trimester.^28^ Half of the women in this study did not commence CGM until the second trimester, indicating the importance of culturally safe systems, which include access to preconception care and early referrals for timely pregnancy care.^5,7^ Proposed CGM targets were not associated with LGA, and perhaps determining a target for average glucose should be considered. We did not find any association with CV, which suggests that the level of hyperglycemia may be more relevant than glucose variability for LGA risk in women with T2DM.

Larger studies are needed to explore these observations further, and to confirm whether CGM metrics demonstrate an association with LGA that is independent of other confounders such as maternal obesity. There is a linear relationship between increasing HbA1c levels and LGA risk.^32^ Our findings suggest that a GMI and an HbA1c target of <6.1% (<43 mmol/mol) in the third trimester may reduce the risk of LGA in T2DM pregnancy.

The limitations of this study include its small sample size, which limited our ability to adjust for multiple confounders, the inherent problems of using a first-generation sensor, and variable sensor use dependent on scanning. Average sensor activity time was 60%, which may have affected the accuracy of the CGM metrics. We did not assess potential confounders such as aspirin or ascorbic acid use, which may affect CGM accuracy,^34^ or intrapartum glycemic control, which may impact neonatal hypoglycemia risk.^35^ We had limited data from the first trimester, and hence, data analysis for early pregnancy metrics included both first and second trimester data. This limits trimester-specific conclusions about associations with neonatal outcomes and underestimated improvements in glucose metrics, which may have occurred between the first and second trimesters.

It is possible that if individual metrics were analyzed for more than 2 weeks, associations may become more evident, and this should be confirmed in future studies. Nevertheless, the findings from this study add valuable information about a population that is distinct from urban Europid women included in previous studies and the first to describe glucose metrics for a high-risk multiethnic population of pregnant women with T2DM.

This study was driven by the need to address diabetes management for this group of predominantly Aboriginal and Torres Strait Islander women living in regional and remote settings, with multiple comorbidities such as high BMI, smoking, alcohol use, hypertension, and high rates of neonatal complications. The findings are particularly generalizable to other high-risk populations with similar challenging clinical and social circumstances, who require intensive individualized treatment to optimize outcomes for mothers and future generations. Ensuring appropriate staffing to support early referral and maintenance of CGM use throughout pregnancy are essential to the successful use of and engagement with CGM technology.^7^ Evaluation of strategies to improve access to preconception planning, early antenatal care, and health resourcing to optimize culturally appropriate diabetes management is critical, and is being undertaken in northern Australia.^5,20,36^

These data provide important information for planning future randomized trials, which are necessary to examine the clinical efficacy of CGM and to clarify treatment targets for T2DM in pregnancy. Although some limitations of reduced sensor activity time and accuracy may be improved by newer generation CGM devices, discontinuation and suboptimal wear time will impact any CGM device used. It will be important to expand on previous acceptability data^7,8^ and include qualitative information on the perspectives and attitudes of women with T2DM to using CGM in future studies.

In conclusion, in this high-risk population of women with T2DM pregnancy, evaluation of CGM metrics revealed limited improvement in glycemic levels from early to late pregnancy. Yet, improvement was greater for women who were able to use sensors more consistently. Neonatal hypoglycemia was associated with all CGM metrics and CGM targets except TBR, whereas LGA was associated with hyperglycemia metrics in early pregnancy. Future studies are required to assess whether CGM technology interventions starting in early pregnancy can reduce the risk of LGA and other neonatal complications. It is imperative that new approaches to diabetes management for women with T2DM, particularly those from culturally and linguistically diverse backgrounds, are established in partnership with women and their communities to improve outcomes for women and their offspring.

## Supplementary Material

Supplemental data

Supplemental data

Supplemental data

Supplemental data

Supplemental data

Supplemental data

Supplemental data
