# Supplementary material for: Continuous Glucose Monitoring Metrics in High-Risk Pregnant Women with Type 2 Diabetes
Source: Diabetes Technol Ther. 2023 Nov 23;25(12):836–44. doi: 10.1089/dia.2023.0300 (PMC10698759; doi:10.1089/dia.2023.0300)
Supplement: Supplemental data [file Suppl_TableS1.docx]

**Supplemental Table 1:** **Maternal characteristics, pregnancy outcomes and CGM use, in those with and without Neonatal Hypoglycaemia and LGA**

|  | **Total**  n=41 | **Neonatal hypoglycaemia**  n=21 (51%) | **No Neonatal hypoglycaemia**  n=20 (49%) | **LGA**  n=23 (56%) | **No LGA**  n=18 (44%) |
| --- | --- | --- | --- | --- | --- |
| ***Maternal characteristics*** |  |  |  |  |  |
| Age, years | 33.2 (5.5) | 32.9 (5.1) | 33.4 (6.0) | 32.6 (5) | 33.9 (5) |
| 1^st^ trimester BMI, kg/m^2^ | 32.8 (5.9) | 33.5 (6.1) | 32.1 (5.7) | **35.8 (5)** | **29.4 (5)** |
| 1^st^ trimester HbA1c, % | 7.8 (6.6,9.1) | **8.3 (7.6, 9.2)** | **6.5 (5.8, 8.0)** | 8.1 (6.9, 9.1) | 7.6 (5.4, 9.2) |
| Remote Locality, n (%) | 13 (32%) | 8 (38%) | 5 (25%) | 8 (34%) | 5 (28%) |
| Aboriginal or Torres Strait Islander Ethnicity^1^,n(%) | 30 (73%) | 16 (76%) | 14 (70%) | 18 (78%) | 12 (67%) |
| Nulliparity, n (%) | 6 (15%) | 4 (19%) | 2 (10%) | 2 (8.7%) | 4 (22%) |
| Pre-existing Hypertension, n (%) | 8 (33%) | 5(33%) | 3 (33%) | 3 (27%) | 5 (38%) |
| Smoking, n (%) | 17 (43%) | **12 (57%)** | **5 (26%)** | 10 (43%) | 7 (41%) |
| Alcohol, n (%) | 6 (16%) | **5 (26%)** | **1 (6%)** | 3 (15%) | 3(18%) |
| Time since diagnosis <5y, n (%) | 15 (50%) | 5 (31%) | 10 (71%) | 7 (50%) | 10 (50%) |
| Insulin use, n (%) | 37 (90%) | **21 (100%)** | **16 (80%)** | **23 (100%)** | **14 (78%)** |
| Insulin dose per day, units | 89 (78) | 109 (76) | 66 (76) | 112 (83) | 59 (62) |
| Metformin use, n (%) | 27 (66%) | 15 (71%) | 12 (60%) | 16 (70%) | 11 (61%) |
| ***CGM use*** |  |  |  |  |  |
| Weeks of sensor wear, weeks | 15.4 (7.8) | 15.4 (7.5) | 15.3 (8.8) | 14.3 (7) | 16.6 (9) |
| Sensor activity time in early pregnancy^2^, % | 63 (23) | 56 (25) | 70 (20) | **56 (22)** | **71 (23)** |
| Sensor activity time in late pregnancy^2^, % | 62 (20) | 64 (23) | 61 (18) | 64 (19) | 60 (22) |
| Number of scans per day in early pregnancy | 4.3 (4.4) | 4.2 (5.6) | 4.4 (2.5) | 3.2 (2.7) | 5.7 (5.6) |
| Number of scans per day in late pregnancy | 4.4 (3.1) | 4.9 (3.4) | 3.8 (2.9) | 4.3 (2.8) | 4.5 (3.6) |
| Gestation first used, weeks | 16.0 (7.0) | 15.7 (7.4) | 15.8 (6.4) | 15.3 (6.3) | 16.8 (7.8) |
| Gestation last used, weeks | 35.6 (2.2) | 35.5 (1.8) | 35.6 (2.7) | 35.0 (2.5) | 36.3 (1.5) |
| ***Pregnancy outcomes*** |  |  |  |  |  |
| Prematurity, n (%) | 14 (34%) | **11 (52%)** | **3 (15%)** | 8 (35%) | 6 (33%) |
| Gestational age at birth, weeks | 37.4  (36, 38) | 36.1 (35.5, 37.7) | 37.5  (37.1, 38.4) | 37.4  (35.8, 37.8) | 37.2  (35.1, 38.1) |
| Birth weight, g | 3497(779) | 3647 (871) | 3340 (655) | 3961 (607) | 2905 (538) |
| Caesarean section, n (%) | 27 (66%) | 16 (76%) | 11 (55%) | 18 (78%) | 9 (50%) |
| Pre-eclampsia | 8 (31%) | 4 (31%) | 4 (31%) | 5 (42%) | 2 (21%) |
| LGA >90^th^ percentile, n(%) | 23 (56%) | **15 (71%)** | **8 (40%)** | - | - |
| Neonatal hypoglycaemia, n(%) | 21 (51%) | - | - | 15 (65%) | 6 (33%) |
| Neonatal hospital stay,days | 3 (2, 13) | **7 (3, 19)** | **2 (1, 4)** | 3 (2, 14) | 3.5 (1, 6) |
| Respiratory distress, n (%) | 15 (37%) | **13 (62%)** | **2 (11%)** | 11 (50%) | 4 (22%) |

Data are presented as mean (SD) or median (interquartile range). Data in **bold** have significance p<0.05. Total n is less for the following characteristics: 1^st^ trimester HbA1c, n=30; pre-existing hypertension n=24; alcohol use n=37; time since T2DM diagnosis n=30; sensor activity time and average scans per day in early pregnancy, n=38, pre-eclampsia n=26.

^1.^Ethnicity is self-reported. Other ethnicities included Indian (n=4), African (n=2), Caucasian (n=1), Filipino (n=1), Pacific Islander (n=1), not specified (n=2)

^2.^Sensor activity time is the percent of time with CGM output over a 14 day period.
