# Supplementary material for: Continuous Glucose Monitoring Metrics in High-Risk Pregnant Women with Type 2 Diabetes
Source: Diabetes Technol Ther. 2023 Nov 23;25(12):836–44. doi: 10.1089/dia.2023.0300 (PMC10698759; doi:10.1089/dia.2023.0300)
Supplement: Supplemental data [file Suppl_FigureS1.docx]

**Supplemental Figure 1: CGM glucose management indicator (GMI), average glucose and coefficient of variation (CV) in early vs. late pregnancy**

| 1a. CGM metrics in total cohort, n=41 |  |  |
| --- | --- | --- |
|  |  |  |
| 1b. CGM metrics if sensor activity time >50%,n=29 |  |  |
|  |  |  |

Figure 1a. The median GMI% (IQR) was 6.2 (5.7-6.5) vs. 6.1 (5.7-6.3), p=0.36; mean average glucose mmol/L (SD) 7.0 (1.9) vs. 6.5 (1.4), mg/dL 126 (34) vs. 117 (25), p=0.12; CV% 28.3 (6.9) vs. 30.5 (6.2), p=0.07

Figure 1b. If sensor activity time >50%, Median GMI 6.1 (5.6-6.5) vs. 5.9 (5.6-6.2), p=0.21, Average glucose in mmol/L 7.1 (1.9) vs. 6.1 (1.1), in mg/dL 128 (32) vs. 110 (20), p=0.005; CV% 27.9 (7.3) vs. 31.4 (6.3), p=0.01

***** Difference between early and late values p<0.05

Definitions: Early pregnancy, first 2 weeks of sensor use, mean gestation 16 weeks, range 6-28 weeks. Late pregnancy, last 2 weeks of sensor use, mean gestation 35 weeks, range 28-38 weeks.
