# Supplementary material for: Continuous Glucose Monitoring Metrics in High-Risk Pregnant Women with Type 2 Diabetes
Source: Diabetes Technol Ther. 2023 Nov 23;25(12):836–44. doi: 10.1089/dia.2023.0300 (PMC10698759; doi:10.1089/dia.2023.0300)
Supplement: Supplemental data [file Suppl_FigureS2.docx]

**Supplemental Figure 2: Change in Time in Range % from Early to Late pregnancy for Individuals**

Early pregnancy TIR: mean (range) gestation 16 (6 to 28) weeks. Late pregnancy TIR: mean (range) gestation 35 (28 to 38) weeks.
