# Supplementary material for: Continuous Glucose Monitoring Metrics in High-Risk Pregnant Women with Type 2 Diabetes
Source: Diabetes Technol Ther. 2023 Nov 23;25(12):836–44. doi: 10.1089/dia.2023.0300 (PMC10698759; doi:10.1089/dia.2023.0300)
Supplement: Supplemental data [file Suppl_TableS4.docx]

**Supplemental Table 4: Trimester specific HbA1c levels for neonatal hypoglycaemia and large for gestational age (LGA) groups**

| **Laboratory HbA1c** | **Total**  n=41 | **Neonatal hypoglycaemia** n=21 (51%) | **No Neonatal hypoglycaemia** n=20 (49%) | P value | **LGA**  n=23 (56%) | **No LGA**  n=18 (44%) | P value |
| --- | --- | --- | --- | --- | --- | --- | --- |
|  |  |  |  |  |  |  |  |
| 1^st^ trimester HbA1c, % | 7.8 (6.6, 9.1) | **8.3 (7.6, 9.2)** | **6.5 (5.8, 8.0)** | **0.01** | 8.1 (6.9, 9.1) | 7.6 (5.4, 9.2) | 0.42 |
| 2^nd^ trimester HbA1c, % | 6.3 (5.4, 7.1) | **7.6 (7.1, 8.1)** | **5.7 (5.4, 6.3)** | **0.01** | 6.55 (6.3, 7.1) | 5.5 (5.3, 6.7) | 0.15 |
| 3^rd^ trimester HbA1c, % | 6.8 (6.1, 7.4) | **6.9 (6.3, 8.1)** | **6.3 (5.8, 7.1)** | **0.04** | 6.8 (6.5, 7.7) | 6.1 (5.8, 7.1) | 0.05 |
| 1^st^ trimester HbA1c <6.5%, n (%) | 6 (20%) | **0 (0%)** | **6 (50%)** | **0.001** | 3 (16%) | 3 (27%) | 0.44 |
| 3^rd^ trimester HbA1c <6.1%, n (%) | 8 (23%) | **1 (6%)** | **7 (39%)** | **0.02** | **1 (6%)** | **7 (41%)** | **0.01** |

Data are presented as median (interquartile range) or n (%)
