# Supplementary material for: Continuous Glucose Monitoring Metrics in High-Risk Pregnant Women with Type 2 Diabetes
Source: Diabetes Technol Ther. 2023 Nov 23;25(12):836–44. doi: 10.1089/dia.2023.0300 (PMC10698759; doi:10.1089/dia.2023.0300)
Supplement: Supplemental data [file Suppl_TableS2.docx]

**Supplemental Table 2. Adjusted associations of CGM metrics and HbA1c with neonatal hypoglycaemia**

| **Glucose metrics** | OR  Unadjusted | 95% CI | OR  Adjusted for BMI | 95% CI | OR  Adjusted for early HbA1c | 95% CI | OR Adjusted for Early TIR | 95% CI |
| --- | --- | --- | --- | --- | --- | --- | --- | --- |
| ***Early pregnancy metrics^a^*** |  |  |  |  |  |  |  |  |
| TIR, % | 0.96 | 0.93, 1.00 | 0.96 | 0.92, 1.00 | 0.98 | 0.94, 1.01 | - | - |
| TAR, % | 1.04 | 0.99, 1.06 | 1.03 | 0.99, 1.06 | 1.02 | 0.99, 1.05 | - | - |
| TBR, % | 0.93 | 0.83, 1.05 | 0.94 | 0.83, 1.06 | 0.91 | 0.74, 1.09 | - | - |
| Average glucose, mmol/L | **1.67** | **1.01, 2.78** | **1.67** | **1.01, 2.79** | 1.48 | 0.85, 2.58 | 1.75 | 0.63, 5.02 |
| GMI, % | 3.07 | 0.92, 10.2 | 3.17 | 0.90, 11.1 | 2.61 | 0.68, 9.90 | 2.74 | 0.68, 11.5 |
| SD, mmol/L | **4.91** | **1.26, 19.0** | **5.07** | **1.33, 19.2** | 2.76 | 0.63, 12.0 | 3.82 | 0.76, 19.1 |
| CV% | 1.07 | 0.97, 1.18 | 1.08 | 0.98, 1.19 | 1.03 | 0.93, 1.16 | 1.10 | 0.97, 1.26 |
| Interquartile range | **26.7** | **2.41, 293** | **98.5** | **2.71, 348** | 240 | 0.31, 478 | **34.8** | **2.41, 487** |
| Early HbA1c, % | **1.61** | **1.01, 2.54** | 1.51 | 0.96, 2.36 | - | - | 1.41 | 0.89, 2.21 |
| ***Early glycaemic targets^b^*** |  |  |  |  |  |  |  |  |
| TIR >70% | **0.16** | **0.04, 0.67** | **0.13** | **0.02, 0.59** | **0.20** | **0.04, 0.95** | 0.16 | 0.01, 1.43 |
| TAR<25% | **0.15** | **0.03, 0.65** | **0.14** | **0.03, 0.63** | **0.21** | **0.04, 1.00** | 0.14 | 0.01, 1.36 |
| TBR <4% | 1.05 | 0.28, 3.51 | 1.04 | 0.27, 4.24 | 1.15 | 0.26, 5.01 | 0.78 | 0.19, 3.19 |
| CV<36% | 0.85 | 0.16, 4.51 | 0.74 | 0.13, 4.18 | 3.11 | 0.34, 28.2 | 1.10 | 0.17, 6.15 |
| GMI <6.5 | 0.43 | 0.10, 1.79 | 0.42 | 0.06, 2.78 | 0.66 | 0.09, 4.55 | 1.33 | 0.56, 51.4 |
| Early HbA1c <6.5% | - | -- | - | - | - | - |  |  |
| ***Late pregnancy metrics^a^*** |  |  |  |  |  |  |  |  |
| TIR, % | **0.94** | **0.90, 0.99** | **0.95** | **0.90, 0.99** | 0.96 | 0.91, 1.01 | 0.96 | 0.92, 1.00 |
| TAR, % | **1.05** | **1.01, 1.09** | **1.05** | **1.01, 1.09** | 1.03 | 0.98, 1.08 | **1.05** | **1.01, 1.09** |
| TBR, % | 0.98 | 0.90, 1.07 | 0.97 | 0.90, 1.06 | 0.99 | 0.90, 1.08 | 0.92 | 0.81, 1.02 |
| Average glucose, mmol/L | **1.97** | **1.08, 3.61** | **1.97** | **1.07, 3.63** | 1.48 | 0.74, 2.98 | **1.95** | **1.01, 3.75** |
| GMI, % | 3.90 | 0.85, 17.7 | 3.98 | 0.82, 19.1 | 2.90 | 0.45, 18.4 | 3.92 | 0.70, 21.1 |
| SD, mmol/L | **5.64** | **1.47, 21.5** | **8.57** | **1.78, 40.6** | 3.08 | 0.72, 13.2 | **4.21** | **1.07, 16.4** |
| CV% | **1.13** | **1.01, 1.26** | **1.17** | **1.03, 1.33** | 1.09 | 0.97, 1.23 | 1.09 | 0.97, 1.23 |
| Interquartile range, mmol/L | **5.25** | **1.71, 16.1** | **9.28** | **2.03, 42.2** | 2.83 | 0.93, 8.40 | **4.50** | **1.48, 13.6** |
| 3^rd^ trimester HbA1c, % | 2.01 | 0.91, 4.65 | 1.75 | 0.89, 3.43 |  |  |  |  |
| ***Late glycaemic targets^b^*** |  |  |  |  |  |  |  |  |
| TIR >70% | **0.08** | **0.01, 0.41** | **0.05** | **0.01, 0.31** | **0.16** | **0.03, 0.91** | **0.13** | **0.02, 0.66** |
| TAR <25% | **0.11** | **0.02, 0.52** | **0.08** | **0.01, 0.47** | 0.21 | 0.04, 1.06 | **0.14** | **0.02, 0.69** |
| TBR <4% | 2.04 | 0.58, 7.17 | 1.71 | 0.47, 6.12 | 2.34 | 0.57, 9.61 | 3.61 | 0.85, 15.3 |
| CV <36% | **0.18** | **0.03, 0.99** | **0.05** | **0.01, 0.57** | 0.32 | 0.05, 2.04 | 0.34 | 0.05, 2.15 |
| GMI <6.5 | 0.78 | 0.11, 5.33 | 0.85 | 0.11, 6.12 | 3.75 | 0.22, 65.8 | 1.36 | 0.15, 11.4 |
| GMI <6.1 | **0.12** | **0.02, 0.59** | **0.10** | **0.01, 0.53** | **0.19** | **0.03, 0.99** | **0.09** | **0.01, 0.56** |
| 3^rd^ trimester HbA1c <6.1% | **0.09** | **0.01, 0.91** | **0.09** | **0.01, 0.89** | 0.19 | 0.01, 3.13 | 0.12 | 0.01, 1.33 |

Supplemental table 1 definitions: Data are odds ratio (OR) and 95% confidence intervals (CI). ^a^ OR per unit increase; ^b^ OR for meeting target. Early metrics, first 2 weeks of sensor use, mean (range) gestation 16 (6 to 28) weeks. Late metrics, last 2 weeks of sensor use, mean (range) gestation 35 (28 to 38) weeks. Early HbA1c, 1^st^ or 2^nd^ trimester HbA1c, mean (SD) 9.6 (6) weeks gestation. TIR, time in range; TAR, time above range; TBR, time below range; CV, glucose coefficient of variation; SD, glucose standard deviation; GMI, glucose management indicator. Glucose target range defined as glucose 3.5-7.8 mmol/L (63-140 mg/dL) and TIR/TAR/TBR expressed as a percentage of all time CGM is active over a 14-day period.
