# Supplementary material for: Continuous Glucose Monitoring Metrics in High-Risk Pregnant Women with Type 2 Diabetes
Source: Diabetes Technol Ther. 2023 Nov 23;25(12):836–44. doi: 10.1089/dia.2023.0300 (PMC10698759; doi:10.1089/dia.2023.0300)
Supplement: Supplemental data [file Suppl_TableS3.docx]

**Supplemental Table 3.** **Adjusted associations of CGM metrics and HbA1c with large for gestational age**

| **Glucose metrics** | OR  Unadjusted | 95%CI | OR  Adjusted for BMI | 95%CI | OR  Adjusted for  Early HbA1c | 95% CI | OR  Adjusted for  Early TIR | 95% CI |
| --- | --- | --- | --- | --- | --- | --- | --- | --- |
| ***Early pregnancy metrics^a^*** |  |  |  |  |  |  |  |  |
| TIR, % | **0.96** | **0.92, 0.99** | **0.91** | **0.84, 0.98** | 0.96 | 0.93, 1.00 | **-** |  |
| TAR, % | **1.04** | **1.01, 1.08** | **1.08** | **1.01, 1.16** | 1.03 | 0.98, 1.08 | **-** |  |
| TBR, % | 0.91 | 0.80, 1.04 | 0.88 | 0.73, 1.08 | 0.85 | 0.66, 1.04 | - |  |
| Average glucose, mmol/L | **1.84** | **1.04, 3.28** | **3.07** | **1.19, 7.89** | 1.81 | 0.97, 3.39 | 1.61 | 0.59, 4.33 |
| GMI, % | 2.58 | 0.83, 7.91 | 4.36 | 0.81, 23.5 | 1.61 | 0.44, 5.87 | 1.21 | 0.23, 6.31 |
| SD, mmol/L | 2.07 | 0.68, 6.21 | 4.11 | 0.98, 17.2 | 1.23 | 0.33, 4.49 | 0.78 | 0.17, 3.55 |
| CV% | 0.99 | 0.91, 1.09 | 1.00 | 0.90, 1.12 | 0.93 | 0.83, 1.05 | 0.98 | 0.87, 1.09 |
| Interquartile range | **6.48** | **1.36, 30.7** | **18.0** | **1.12, 290** | **7.36** | **1.29, 41.9** | 4.98 | 0.93, 26.4 |
| Early HbA1c, % | 1.20 | 0.84, 1.71 | 1.19 | 0.81, 1.73 | - | - | 1.13 | 0.77, 1.65 |
| ***Early glycaemic targets^b^*** |  |  |  |  |  |  |  |  |
| TIR >70% | 0.52 | 0.14, 1.91 | **0.08** | **0.01, 0.78** | 0.73 | 0.17, 3.05 | 7.81 | 0.55, 109 |
| TAR<25% | 0.31 | 0.08, 1.21 | **0.06** | **0.01, 0.61** | 0.41 | 0.09, 1.87 | 1.39 | 0.15, 12.4 |
| TBR <4% | 1.26 | 0.33, 4.70 | 1.10 | 0.19, 6.11 | 0.92 | 0.22, 3.80 | 0.92 | 0.22, 3.80 |
| CV<36% | 2.01 | 0.37, 10.5 | 0.97 | 0.11, 8.61 | 9.58 | 0.78, 111 | 3.61 | 0.52, 24.9 |
| GMI <6.5 | 0.46 | 0.07, 2.85 | 0.07 | 0.01, 1.33 | 0.77 | 0.04, 14.2 | 10.9 | 0.43, 274 |
| ***Late pregnancy metrics^a^*** |  |  |  |  |  |  |  |  |
| TIR, % | 0.98 | 0.95, 1.01 | 0.98 | 0.95, 1.02 | 1.01 | 0.96, 1.06 | 0.98 | 0.95, 1.02 |
| TAR, % | 1.02 | 0.98, 1.05 | 1.02 | 0.98, 1.05 | 1.01 | 0.96, 1.05 | 1.01 | 0.98, 1.04 |
| TBR, % | 0.94 | 0.85, 1.03 | 0.92 | 0.82, 1.04 | 0.94 | 0.85, 1.03 | 0.96 | 0.85, 1.07 |
| Average glucose, mmol/L | 1.57 | 0.91, 2.69 | 1.49 | 0.80, 2.78 | 1.48 | 0.75, 3.13 | 1.37 | 0.79, 2.38 |
| GMI, % | 3.35 | 0.73, 15.3 | 3.08 | 0.52, 18.2 | 2.36 | 0.36, 15.0 | 2.06 | 0.40, 10.3 |
| SD, mmol/L | 3.39 | 0.99, 11.5 | 3.25 | 0.70, 15.0 | 2.75 | 0.66, 11.5 | 2.81 | 0.76, 10.4 |
| CV% | 1.13 | 0.96, 1.22 | 1.05 | 0.93, 1.20 | 1.04 | 0.89, 1.18 | 1.06 | 0.94, 1.20 |
| Interquartile range, mmol/L | **2.29** | **1.02, 5.10** | 2.28 | 0.83, 6.23 | 2.07 | 0.86, 4.14 | 2.03 | 0.89, 4.62 |
| 3^rd^ trimester HbA1c, % | 1.59 | 0.83, 3.05 | 1.78 | 0.82, 3.86 | 1.68 | 0.75, 3.82 | 1.11 | 0.52, 2.36 |
| ***Late glycaemic targets^b^*** |  |  |  |  |  |  |  |  |
| TIR >70% | 0.54 | 0.15, 1.92 | 0.52 | 0.10, 2.56 | 1.07 | 0.23, 5.02 | 0.58 | 0.13, 2.53 |
| TAR <25% | 0.31 | 0.08, 1.12 | 0.32 | 0.05, 1.93 | 0.39 | 0.07, 1.99 | 0.34 | 0.07, 1.54 |
| TBR <4% | 1.44 | 0.41, 5.03 | 1.56 | 0.33, 7.37 | 1.59 | 0.41, 6.03 | 1.07 | 0.26, 4.35 |
| CV <36% | 0.80 | 0.18, 3.44 | 0.40 | 0.05, 2.82 | 1.50 | 0.30, 7.52 | 1.08 | 0.18, 6.54 |
| GMI <6.1 | **0.21** | **0.04, 0.94** | 0.18 | 0.02, 1.19 | 0.34 | 0.06, 1.74 | 0.31 | 0.06, 1.65 |
| GMI <6.5 | 0.33 | 0.03, 3.37 | 0.54 | 0.04, 6.62 | 0.77 | 0.04, 14.7 | 0.49 | 0.04, 6.10 |
| 3^rd^ trimester HbA1c <6.1% | **0.08** | **0.01, 0.78** | **0.04** | **0.01, 0.71** | **0.08** | **0.01, 0.89** | 0.13 | 0.01, 1.50 |

Data are odds ratio (OR) and 95% confidence intervals (CI). ^a^ OR per unit increase; ^b^ OR for meeting target. Early metrics, first 2 weeks of sensor use, mean (range) gestation 16 (6 to 28) weeks. Late metrics, last 2 weeks of sensor use, mean (range) gestation 35 (28 to 38) weeks. Early HbA1c, 1^st^ or 2^nd^ trimester HbA1c, mean (SD) 9.6 (6) weeks gestation. TIR, time in range; TAR, time above range; TBR, time below range; CV, glucose coefficient of variation; SD, glucose standard deviation; GMI, glucose management indicator. Glucose target range defined as glucose 3.5-7.8 mmol/L (63-140 mg/dL) and TIR/TAR/TBR expressed as a percentage of all time CGM is active over a 14-day period.
