# Supplementary material for: Continuous Glucose Monitoring Metrics in High-Risk Pregnant Women with Type 2 Diabetes
Source: Diabetes Technol Ther. 2023 Nov 23;25(12):836–44. doi: 10.1089/dia.2023.0300 (PMC10698759; doi:10.1089/dia.2023.0300)
Supplement: Supplemental data [file Suppl_FigureS3.docx]

**Supplemental Figure 3. Absolute difference between finger-stick glucose and scanned glucose measurements***

*Data are 573 paired scanned and finger-stick capillary glucose measurements by women using the Freestyle Libre 1 glucometer.

The mean Absolute Relative Difference (ARD) was 16.7% and median ARD was 13.6%.

At glucose ranges under 4mmol/L (n=50), between 4 to 7.8mmol/L (n=415) and above 7.8mmol/L (n=108) the mean ARD was 14.4%, 17.0%, 16.5% and median ARD was 10.5%, 14.2% and 10.1%, respectively.
